# Muscle architecture and morphology as determinants of explosive strength

**DOI:** 10.1007/s00421-020-04585-1

**Published:** 2021-01-17

**Authors:** T. M. Maden-Wilkinson, T. G. Balshaw, G. J. Massey, J. P. Folland

**Affiliations:** 1 Physical Activity, Wellness and Public Health Research Group, Department of Sport and Physical Activity, Sheffield Hallam University, Sheffield, UK; 2 Versus Arthritis Centre for Sport, Exercise and Osteoarthritis Research, Loughborough University, Leicestershire, UK; 3 School of Sport, Exercise, and Health Sciences, Loughborough University, Leicestershire, UK

**Keywords:** Explosive strength, Magnetic resonance imaging, Quadriceps, Muscle architecture

## Abstract

**Purpose:**

Neural drive and contractile properties are well-defined physiological determinants of explosive strength, the influence of muscle architecture and related morphology on explosive strength is poorly understood. The aim of this study was to examine the relationships between *Quadriceps* muscle architecture (pennation angle [Θ_P_] and fascicle length [F_L_]) and size (e.g., volume; Q_VOL_), as well as patellar tendon moment arm (PT_MA_) with voluntary and evoked explosive knee extension torque in 53 recreationally active young men.

**Method:**

Following familiarisation, explosive voluntary torque at 50 ms intervals from torque onset (T_50_, T_100_, T_150_), evoked octet at 50 ms (8 pulses at 300-Hz; evoked T_50_), as well as maximum voluntary torque, were assessed on two occasions with isometric dynamometry. B-mode ultrasound was used to assess Θ_P_ and F_L_ at ten sites throughout the quadriceps (2–3 sites) per constituent muscle. Muscle size (Q_VOL_) and PT_MA_ were quantified using 1.5 T MRI.

**Result:**

There were no relationships with absolute early phase explosive voluntary torque (≤ 50 ms), but θ_P_ (weak), Q_VOL_ (moderate to strong) and PT_MA_ (weak) were related to late phase explosive voluntary torque (≥ 100 ms). Regression analysis revealed only Q_VOL_ was an independent variable contributing to the variance in T_100_ (34%) and T_150_ (54%). Evoked T_50_ was also related to Q_VOL_ and θ_P._ When explosive strength was expressed relative to MVT there were no relationships observed.

**Conclusion:**

It is likely that the weak associations of θ_P_ and PT_MA_ with late phase explosive voluntary torque was via their association with MVT/Q_VOL_ rather than as a direct determinant.

## Introduction

Explosive strength can be defined as the ability to increase force or torque as quickly as possible during a rapid voluntary contraction from a low or resting level (Maffiuletti et al. 2016). There has been a growing appreciation of the functional significance of explosive strength, particularly in situations where the time to generate torque is limited; for instance in sprinting and jumping (Weyand et al. 2010; Tillin et al. 2013b) and during injury-related situations (e.g., anterior cruciate ligament tears within ≤ 50 ms after landing; (Krosshaug et al. 2007; Koga et al. 2010)). In addition explosive strength appears to be important for balance, including responding to perturbations and the avoidance of falls in older adults (Izquierdo et al. 1999; Pijnappels et al. 2008; Bento et al. 2010; Behan et al. 2018), and has been related to subjective and objective measures of function in musculoskeletal patients (e.g., osteoarthritis; Maffiuletti et al. 2010; Winters and Rudolph 2014; Hsieh et al. 2015)). Explosive strength, particularly during the early phase of contraction (0–50 ms) is highly variable between individuals (Folland et al. 2014) which may have implications for all of these functional situations. Moreover, the functional importance of explosive strength highlights the need to understand its physiological determinants.

Research has shown the importance of rapid neuromuscular activation for explosive strength, particularly during the early phase of contraction (i.e., strong positive correlations in the first 50 ms, (de Ruiter et al. 2006, 2007; Folland et al. 2014; DelVecchio et al. 2019)), but the intrinsic contractile properties have also been found to increasingly contribute to the explained variance throughout the rising force–time curve. Specifically: evoked twitch force explained up to 40% of the variance in early phase explosive strength (0–50 ms; (Andersen and Aagaard 2006; Folland et al. 2014); evoked octet force (a train of eight supramaximal pulses at 300 Hz, which drives the muscle at its maximum possible rate of force development [RFD]) accounted for 68% of the variance during the fastest phase of voluntary force development (RFD 50–100 ms; (Folland et al. 2014)); and maximum strength explained 52–90% of the variance in late phase explosive strength (≥ 100 ms (Andersen and Aagaard 2006; Folland et al. 2014)). However, these measures of contractile function (evoked twitch and octet, and maximum strength) represent the summation of various musculoskeletal factors, including muscle size, architecture (fascicle length [F_L_], pennation angle [θ_P_]) and moment arm, which therefore precludes conclusions about the importance of these specific individual factors for explosive strength.

Surprisingly, the potential relationships between explosive strength and muscle architecture variables (F_L_ and θ_P_) are relatively unexplored. There are some theoretical possibilities for how muscle architecture could influence explosive strength. First, a greater θ_P_ has been associated with more fascicle rotation (increase θ_P_) during contraction, and thus greater ‘gearing’ of muscle-shortening velocity to fascicle velocity that constrains fascicle shortening velocity thereby permitting greater force production (4, 16). As fibre rotations occur predominantly at low force (4) θ_P_ might be expected to influence early phase rapid force development. Second, F_L_ is considered reflective of muscle fibre length (Lieber and Friden 2000) and thus serial sarcomere number; that largely dictates the muscle’s maximal shortening velocity (Bodine et al. 1982; Wickiewicz 1984; Lieber and Friden 2000). Under isometric conditions a muscle with longer fascicles and thus a higher shortening velocity may be expected to develop torque more quickly by taking up the inherent compliance present within the in-series force transmitting structures (Edman and Josephson 2007). Despite these theoretical possibilities and other speculations in the literature (Abe et al. 2000; Kumagai et al. 2000; Blazevich et al. 2009a; Bazyler et al. 2017; Wagle et al. 2017) direct experimental evidence examining the association of muscle architecture and explosive strength is lacking. Somewhat related evidence concerns the positive relationship of θ_P_ and maximum isometric strength (*r* = 0.47–0.68) (Wakahara et al. 2013; Strasser et al. 2013; Ando et al. 2015), Given the positive association between maximum strength and late phase explosive strength (Andersen and Aagaard 2006; Folland et al. 2014) this might infer a relationship between θ_P_ and late phase explosive strength. However, the relationship of muscle architecture and explosive strength remains unknown.

The strong relationship between maximum strength and late phase explosive strength (Andersen and Aagaard 2006; Folland et al. 2014) suggests the determinants of maximum strength may also be related to late phase explosive strength. Muscle size (measured either via volume, anatomical cross-sectional area [ACSA] or physiological cross-sectional area [PCSA]) strongly predicts maximum strength (50–90% explained variance: (Bamman et al. 2000; Fukunaga et al. 2001; Blazevich et al. 2009b). Accordingly, we have previously found muscle volume to be related to late phase explosive strength (elbow flexor volume & force at 150 ms *r* = 0.69, (Erskine et al. 2014); knee flexor volume and time to 90 Nm of torque (*r* = − 0.53, (Evangelidis et al. 2017)). However, the comparative importance of the different measures of muscle size for explosive strength has not been explored.

A larger joint moment arm about which muscle forces are applied would be expected to provide a mechanical advantage, transferring given muscle forces into a greater joint torque. To this effect, the Achilles tendon moment arm (*r* = 0.56; (Baxter and Piazza 2014)) and patellar tendon moment arm (*r* = 0.40; Blazevich et al. 2009b; Tresize et al. 2016)) have been related to the isometric strength of the respective muscle groups. However, the relationship of moment arm with explosive strength has yet to be investigated.

Overall, while research has delineated neural and integrated contractile determinants of voluntary explosive strength (Andersen and Aagaard 2006; Folland et al. 2014), the influence of specific musculoskeletal factors, especially muscle architecture remains to be elucidated. The upper bound for any influence of muscle architecture on voluntary explosive strength might be revealed by the relationship of architecture with the purely contractile capacity for rapid force development i.e. evoked explosive strength, that bypasses the voluntary nervous system and drives the muscle at its maximal possible rate of force development (24, 40).

The aim of this study was to assess the relationships between musculoskeletal factors (Quadriceps architecture [F_L_ and θ_P_], size [volume_,_ ACSA, PCSA] and patellar tendon moment arm) and voluntary and evoked explosive strength of the knee extensors. We hypothesised that θ_P_ would be positively associated with voluntary explosive strength throughout the rising torque-time curve, and that F_L_ would be related to early phase explosive strength.

## Materials and methods

### Participants and ethical approval

Fifty-three young men (Age: 25 ± 2 years, Height: 1.75 ± 0.08 m, Body Mass: 71 ± 10 kg) who were healthy, free from musculoskeletal injury, and recreationally active (2160 ± 1309 metabolic equivalent (MET) minutes per week, International Physical Activity Questionnaire short format (Craig et al. 2003), but not involved in any form of systematic physical training in the prior 18 months, were included in this study. The experimental testing procedures were explained to each participant and all participants provided written informed consent before their involvement in this study, which was approved by the Loughborough University Ethical advisory committee and conducted in accordance with the principles of the Declaration of Helsinki.

### Experimental design

Participants completed a familiarisation session, involving practice of all the voluntary contractions performed during subsequent measurement sessions and habituation with evoked (electrically stimulated) contractions, followed by two duplicate neuromuscular measurement sessions separated by 7–10 days. This was done to improve measurement accuracy and minimise the effects of measurement error/noise. Measurement sessions involved a series of unilateral isometric contractions of the knee extensors of the dominant (preferred kicking) leg in the following order: maximum voluntary contractions (MVCs); explosive voluntary contractions; evoked twitch contractions; and evoked octet contractions (second measurement session only). Voluntary and evoked explosive torque measurements were determined from explosive voluntary and evoked octet contractions, respectively. Maximum voluntary torque (MVT) was determined from MVCs. Neuromuscular measurement sessions were performed at a consistent time of the day for each individual participant, and all sessions started between 12:00–19:00 h. Participants were instructed not to participate in strenuous physical activity or consume alcohol for 36 h, and refrain from caffeine consumption for 6 h, before measurement sessions. On a separate occasion, musculoskeletal imaging measurements (2D ultrasonography and MRI) of the quadriceps femoris muscle group were collected. Ultrasonography images were captured at multiple locations along the length of each of the four constituent muscles of the quadriceps femoris (2–3 locations per muscle for a total of ten architecture measurement sites) to provide a comprehensive assessment of fascicle length and pennation angle. Magnetic resonance T1-weighted axial plane images of the thigh and sagittal plane images of the knee joint were acquired to measure muscle size (Q_VOL_, ACSA and PCSA) and patellar tendon moment arm (PT_MA_), respectively. The analysis focused on whole quadriceps measures of size and architecture as the functional outcomes (i.e., maximum and explosive strength), depend on the synergistic and combined actions of the four constituent muscles, and therefore it would seem logical to relate function, which is by nature a combined measure, with overall/combined (averaged) measures of the muscle characteristics.

### Torque measurement

Participants were positioned in an isometric knee extension dynamometer with knee and hip angles of 115° and 126° (180° = full extension), respectively. Previous work from our lab has demonstrated that this knee joint angle is optimal for maximal and voluntary explosive torque production (Lanza et al. 2019). Adjustable straps were tightly fastened across the pelvis and shoulders to prevent extraneous movement. An ankle strap (35 mm width reinforced canvas webbing) was placed ~ 15% of tibial length (distance from lateral malleolus to knee joint space) above the medial malleolus and positioned perpendicular and posterior to the tibia and in series with a calibrated S-Beam strain gauge (Force Logic, Berkshire, UK).The analogue force signal was amplified (× 370; A50 amplifier, Force Logic UK) and sampled at 2000 Hz using an A/D converter (Micro 1401; CED, Cambridge, UK) and recorded with Spike 2 computer software (CED). In offline analysis, force signals were low-pass filtered at 500 Hz using a fourth order zero-lag Butterworth filter (i.e., minimal filtering to facilitate manual determination of explosive contraction onset (Maffiuletti et al. 2016)), gravity corrected by subtracting baseline force, and multiplied by lever length, the distance from the knee joint space to the centre of the ankle strap, to calculate torque.

### Knee extension maximum voluntary contractions

Following a brief warm-up (3 s contractions of 3 × 50, 3 × 75 and 1 × 90% of perceived maximum, with ~ 20 s rest between each), participants performed 3–4 MVCs and were instructed to ‘push as hard as possible’ (knee extension) and rest ≥ 30 s. A horizontal cursor indicating the greatest torque obtained within the session was displayed for biofeedback and verbal encouragement was provided during all MVCs. The highest instantaneous torque recorded during any MVC was defined as MVT.

### Explosive voluntary contractions

Participants performed a series of ten explosive voluntary contractions each separated by 15 s. Participants were instructed to extend their knee ‘as fast and as hard as possible’; with the emphasis on ‘fast’, for 1 s from a relaxed state upon hearing an auditory signal. Contractions involving a visible countermovement or pre-tension were discarded and another attempt made. To indicate if a countermovement or pre-tension had occurred, resting torque was displayed on a sensitive scale. During each explosive contraction participants were required to exceed 80% MVT, which was depicted by an on-screen marker. To provide performance feedback, the time taken to reach 80% MVT was shown after each contraction and the slope of the rising torque-time curve (10 ms time constant) was displayed throughout these contractions with the peak slope of their best attempt indicated with an on-screen cursor. The three best explosive contractions (highest torque at 100 ms and no discernible countermovement or pre-tension, change in baseline force < 0.34 Nm (Equivalent to 1 N accounting for mean lever length) in the preceding 300 ms) were analysed in detail. Contraction onset, during both voluntary explosive and octet contractions, was defined as the last trough before the torque signal permanently deflected away from the envelope of the baseline noise; identified via manual inspection using a systematic standard method by the same trained investigator, in accordance with previously published methods (Tillin et al. 2010). Manual onset detection is considered to provide greater accuracy and reliability than an automatic approach (Tillin et al. 2013a). Briefly, the torque signal was initially viewed with y and x-axis scales of 0.68 Nm and 300 ms, respectively and a vertical cursor placed on torque onset. Accurate placement of the cursor was verified by viewing the signal with a higher resolution. Absolute voluntary explosive torque (averaged across the three best contractions) was quantified at 50 ms intervals from onset to 150 ms (voluntary T_50_, T_100_ and T_150_), and also then expressed relative to MVT (%MVT; relative T_50_, T_100_ and T_150_). Finally, absolute and relative rate of torque development) between sequential time points (0–50 ms [RTD_0–50_], 50–100 ms [RTD_50–100_], and 100–150 ms [RTD_100–150_] were calculated as the ΔTorque/ΔTime (absolute) or Δ%MVT/ΔTime (relative).

### Evoked octet contractions

The femoral nerve was electrically stimulated (constant current, variable voltage stimulator; DS7AH, Digitimer Ltd., UK) with square-wave pulses (0.2 ms duration) to elicit involuntary contractions of the knee extensors whilst the participant was voluntarily passive. Electrical stimuli were applied via a cathode probe (1 cm diameter; Electro Medical Supplies, Wantage, UK) protruding 2 cm perpendicular from the center of a plastic base (4 × 5 cm). The cathode and an anode (carbon rubber electrode, 7 × 10 cm; Electro Medical Supplies, Wantage, UK) were coated with electrode gel and securely taped to the skin over the femoral nerve in the femoral triangle and the greater trochanter, respectively. Twitch contractions (delivery of a single electrical impulse) were conducted first to determine the stimulation intensity for octet contractions. The precise location of the cathode was determined as the position that evoked the greatest twitch response to a submaximal electrical current. Twitch contractions were then elicited at incremental currents (~ 15 s apart) until a simultaneous plateau in peak twitch torque was observed. Thereafter, the electrical current was lowered, and octet stimulation (8 pulses at 300 Hz) was delivered in stepwise increments until the stimulation intensity that elicited twitch force plateau (defined as the maximal stimulation intensity/ current) was reached. Real-time inspection of octet peak torque and peak rate of torque development (10 ms epoch) confirmed a plateau in both variables with incremental stimulation. Subsequently, three supramaximal (120% maximal current) octet contractions were elicited. Absolute octet torque was quantified 50 ms after onset (evoked T_50_) and then expressed relative to MVT (%MVT; relative evoked T_50_). Values recorded from each of the three supra-maximal octet contractions were averaged.

### Muscle architecture

**Fig. 1:**
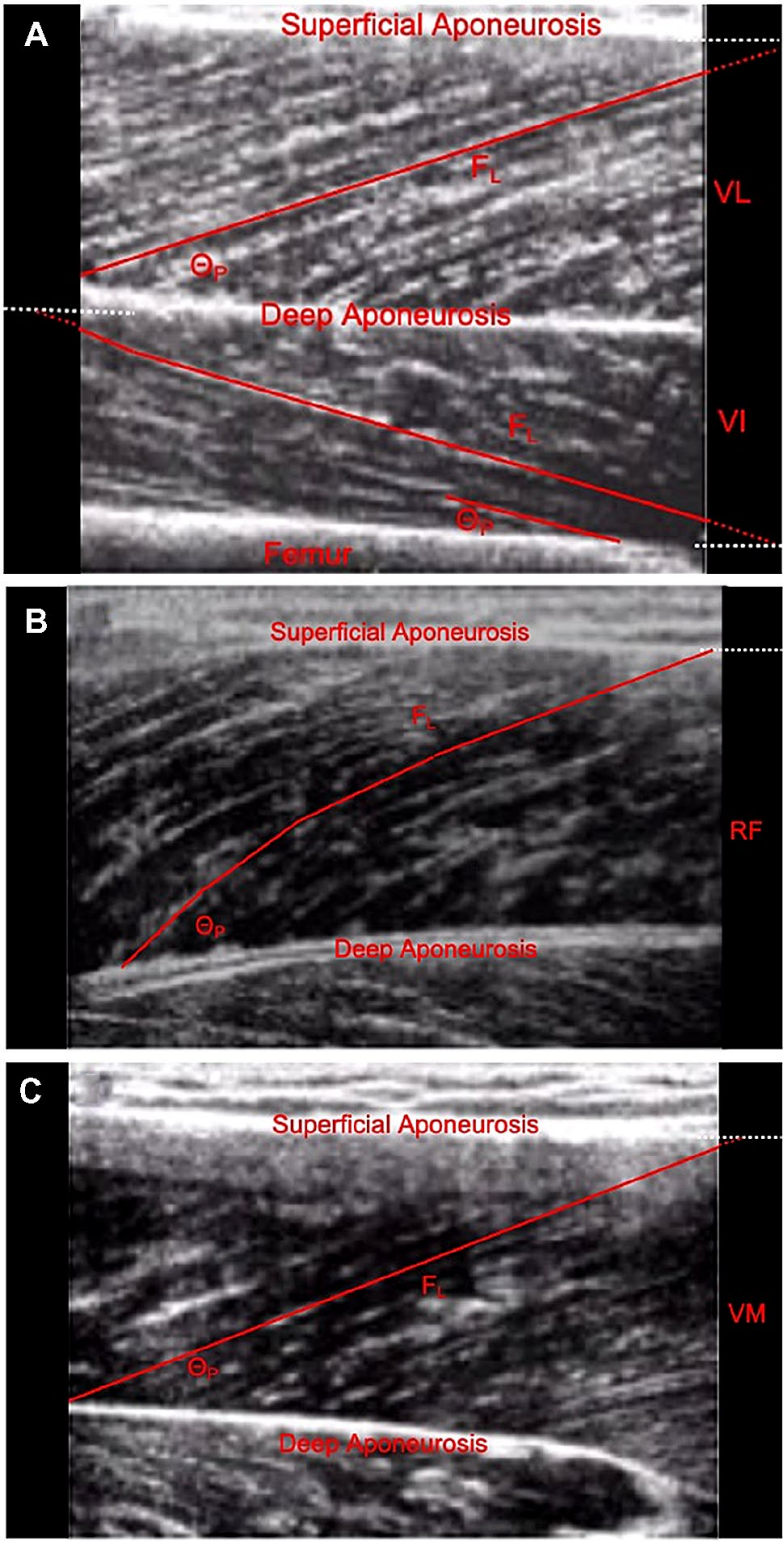
Representative ultrasound images and representation of the architectural assessment sites for **a** Vastus Lateralis (VL) and Intermedius (VI) (50% thigh length; VL_MID_, VI_MID_); **b** Rectus Femoris (RF_MID_) (55% thigh length) and **c** Vastus Medialis (VM) (40% thigh length: VM_PRX_). The dotted red lines show linear extrapolation for the measurement of fascicle length

Muscle architecture of all four quadriceps femoris constituent muscles (VM, VL, VI, and RF) was examined in detail using B-mode ultrasonography (EUB-8500, Hitachi Medical Systems UK Ltd, Northamptonshire, UK) and a 9.2 cm, 5–10 MHz linear-array transducer (EUP-L53L). The participant sat at rest in the same isometric knee extension dynamometer and at the same knee and hip angles as used for strength assessments. Images were captured at rest at 2–3 sites per constituent muscle for a total of ten architecture measurements from each quadriceps. Specific sites were over the mid muscle belly (median longitudinal line, i.e., 50% of superficial medio-lateral width) at the following percentages of thigh length proximal to the knee joint space: VM 20% (VM_DIS_) and 40%(VM_PRX_), VI and VL 25% (VI_DIS_, VL_DIS_), 50% (VI_MID_, VL_MID_) and 75% (VI_PRX_, VL_PRX_), RF 55% (RF_MID_) and 75%(RF_PRX_) (*Fig. **1*), The transducer (coated with water soluble transmission gel) was positioned parallel to the long axis of the thigh (femur), and perpendicular to the skin such that an image with the aponeuroses and the perimysium trajectory of several fascicles was clearly identifiable with no visible fascicle distortion at the edge of the image, and with minimal pressure applied on the dermal surface. Video output from the ultrasound machine was transferred to a computer (via an S-video to USB converter) and images recorded using ez-cap video capture software. Images were later imported into public domain software (Image J, v1.48, National Institutes of Health, Bethesda, USA) for analysis.

Muscle architecture measures were quantified as previously described (Narici 1999). Briefly, pennation angle (Θ_P_) was measured as the angle of insertion of the muscle fibre fascicles into the deep aponeurosis. Muscle Fascicle length was measured as the length of the fascicular path between the insertions into the superficial and deep aponeurosis, where the fascicular path extended beyond the acquired image the missing portion of the fascicle was estimated by extrapolating linearly the fascicular path and the aponeurosis (Narici et al. 2003). Quadriceps Architecture measurements (Θ_P_ and F_L_) taken at multiple sites within each individual muscle were averaged to give a representative value for each muscle, prior to calculating weighted mean quadriceps values (QΘ_P_ and QF_L_) according to proportion of Q_VOL_ of each individual muscle volume_._

### MRI measurement of quadriceps muscle size and patella tendon moment arm

**Fig. 2:**
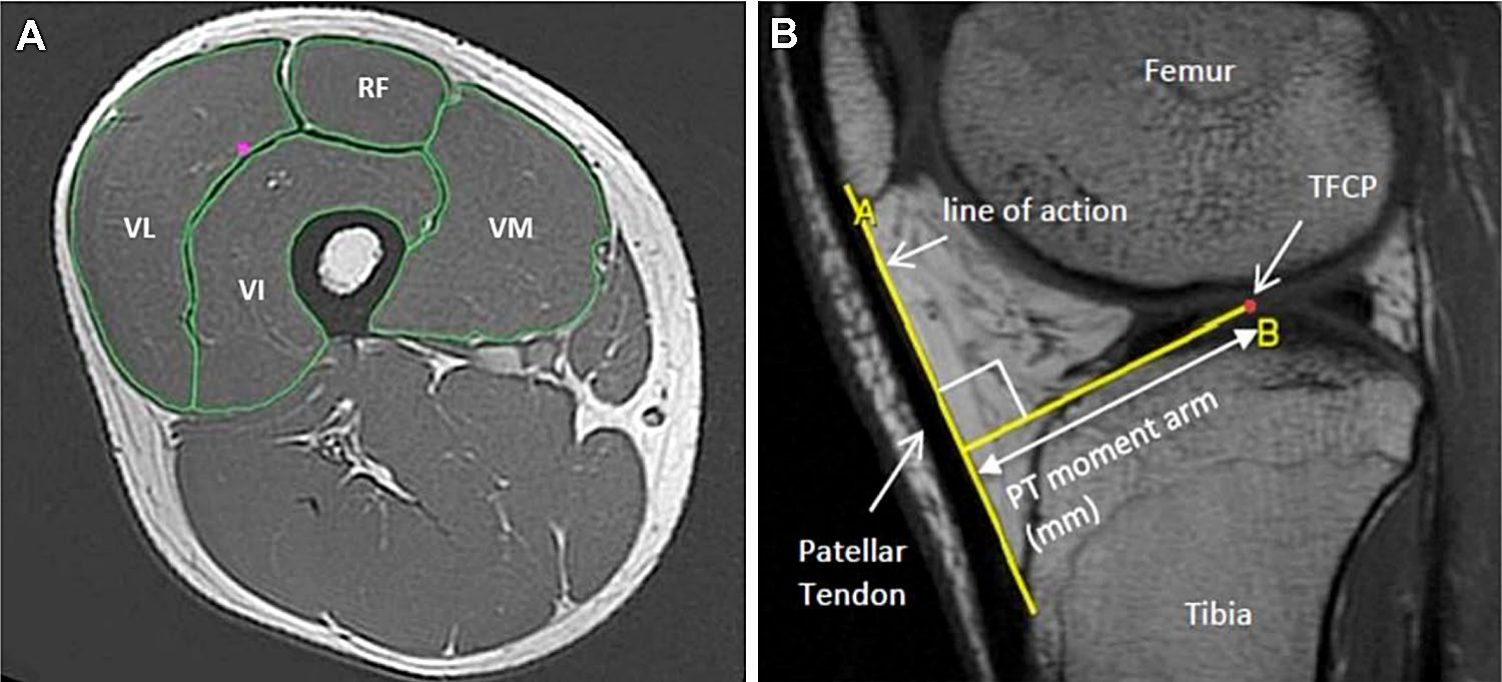
Representative **a** Axial MR image of the thigh and **b** Sagittal MR image of the knee joint. Patellar tendon moment arm was defined as the perpendicular distance between the tendon line of action and the tibio-femoral contact point (TFCP)

Participants reported to the MRI scanner (1.5 T Signa HDxt, GE) having not engaged in strenuous activity in the prior 36 h and were instructed to arrive in a relaxed state having eaten and drunk normally and sat quietly for 15 min prior to their MRI scans. T1-weighted MR images of the dominant leg (thigh and knee) were acquired in the supine position at a knee angle of 163° (due to constraints in knee coil size) and analysed using OsiriX software (Version 6.0, Pixmeo, Geneva, Switzerland). Using a receiver 8-channel whole body coil, axial images (image matrix 512 × 512, field of view 260 × 260 mm, pixel size 0.508 × 0.508 mm, slice thickness 5 mm, inter-slice gap 0 mm) were acquired from the anterior superior iliac spine to the knee joint space in two overlapping blocks. Oil filled capsules placed on the lateral side of the thigh allowed alignment of the blocks during analysis. The quadriceps femoris muscles (vastus lateralis (VL), vastus Intermedius (VI) vastus Medialis (VM), and rectus femoris (RF)) were manually outlined in every third image (i.e., every 15 mm) starting from the most proximal image in which the muscle appeared (Fig. 2a) to assess ACSA along the length of the femur by two investigators. The volume of each muscle was calculated using cubic spline interpolation of the ACSA—femur length plot (GraphPad Prism 6; GraphPad Software) and the sum of these termed total quadriceps volume (Q_VOL_). Effective PCSA was the criterion measure of PCSA and calculated for each muscle as muscle volume divided by mean F_L_ to give PCSA, and then multiplied by the cosine of the mean Θ_P_ (see architecture measurements above for F_L_ and Θ_P_) for Effective PCSA. Q_VOL_ and Effective PCSA (Q_EFF_PCSA) were calculated as the summation of the four individual muscle volumes/Effective PCSAs. QACSA_MAX_ was calculated by the summation of the maximal ACSA from each individual muscle. Inter- and intra-rater reliability for Q_VOL_ calculated from the repeated analysis of five MRI scans was 1.2 and 0.4%, respectively.

Sagittal plane T1-weighted magnetic resonance (1.5 T Signa HDxt; GE) images (2 mm thickness, 0 mm gap) images were used to determine patellar tendon moment arm (PT_MA_) length. PT_MA_ was measured from sagittal plane as the perpendicular distance from the PT line of action to the tibiofemoral contact point, which was the midpoint of the contact distance between the tibia and femur (Fig. 2b).

### Data analysis and statistics

MVT and explosive strength (voluntary and evoked) measurements were averaged over the two testing sessions and these “criterion” values were used in all further analyses. Differences between the individual constituent muscles were assessed with ANOVA and post hoc Bonferroni corrected t-test to determine where any differences lay. The relationships between musculoskeletal variables (Q_VOL_, QACSA_MAX_, Q_EFF_PCSA, Moment arm, F_L_ and Θ_P_) and explosive strength measurements (voluntary and evoked torque, expressed in both absolute terms and relative to MVT) were first assessed with independent Pearson's product moment correlations. Pearson’s product moment correlation *P* values were corrected for multiple tests using the Benjamini–Hochberg procedure (Benjamini and Hochberg 1995) with a false detection rate of 5%, and significance was defined as adjusted *p* < 0.05. Correlation coefficients were considered ‘very weak’ (*r* ≤ 0.30), ‘weak’ (*r* = 0.30–0.50), ‘moderate’ (*r* = 0.50–0.70) and ‘strong’ (*r* = 0.7–0.9) (Moore et al. 2013). In cases where more than one predictor variable showed a corrected significant correlation with the outcome a multiple regression analysis was performed, with only the significant predictors entered into the model. Descriptive statistics are mean ± standard deviation (SD). Variability between subjects for all measures is expressed as coefficient of variation (CV_b_; [Cohort SD/Cohort mean] × 100).

## Results

### Inter-individual variability

**Fig. 3:**
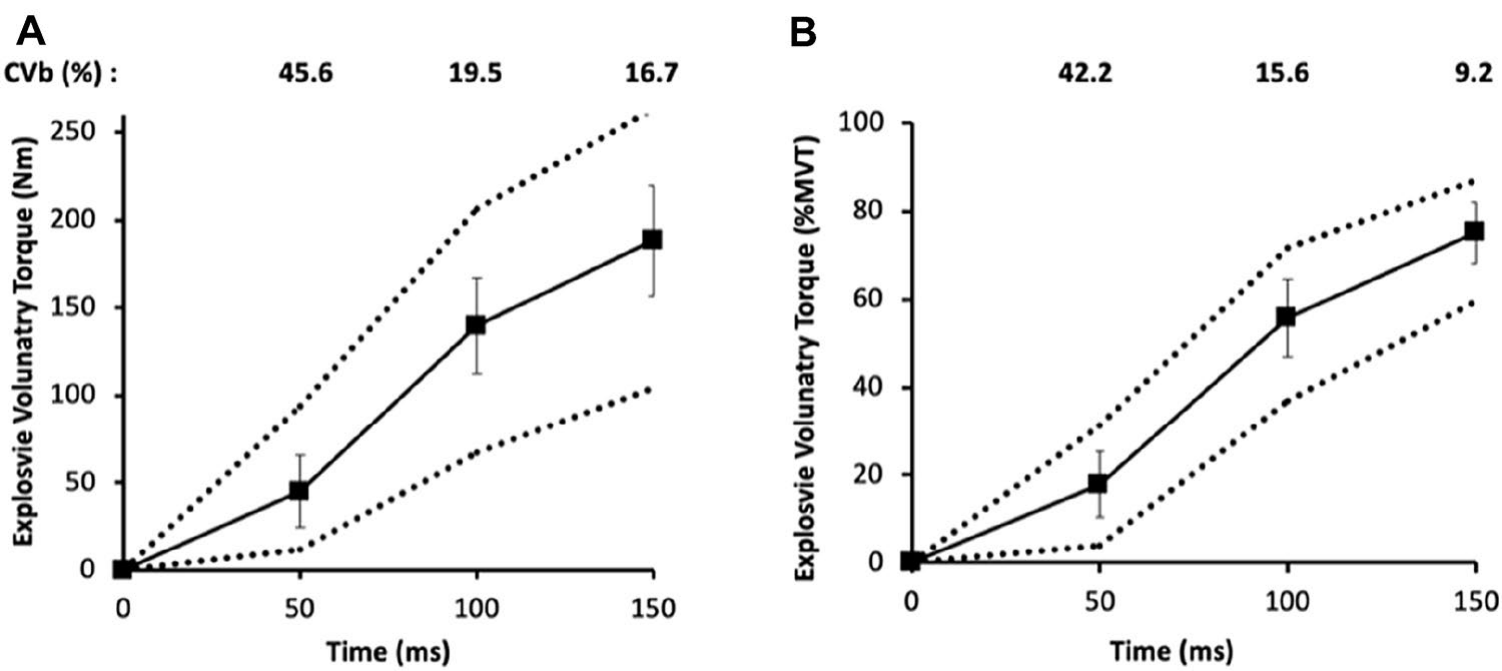
Absolute (**a**) and Relative (**b**, to MVT) torque developed at 50 ms intervals from torque onset during explosive voluntary contractions of the knee extensors. The solid line and squares show mean ± SD (*n* = 53), and the dotted line indicates minimum and maximum values

The between participant variability in voluntary explosive torque was greatest in the early phase of contraction for both absolute (CVb 45.6%, range 11–94 Nm) and relative (CVb 42.2%, range 4–31.3%MVT) T_50_ but decreased as the contractions progressed (Fig. 3). During the octet contractions, CVb of evoked T_50_ was substantially smaller 13% (absolute) and 12% (relative to MVT). There was more modest variability in muscle size indices (Q_VOL_, QACSA_MAX_ and Q_EFF_PCSA CVb 11.2–14.4%) and architecture variables (CVb: QΘ_P_ 10.7%; QF_L_ 9.6%) and low variability for PT_MA_ (CVb 6.7%; Table 1). Maximum voluntary torque was 247 ± 43 Nm (range 173–396 Nm; CVb 17%). There were differences between the size (volume, ACSA_MAX_, _EFF_PCSA), and architecture (Θ_P_ and F_L_) measurements between constituent muscles (Table 2).

**Table 1:** Descriptive statistics for muscle (size and architecture) and joint (moment arm) morphology (*n* = 53)

Variable	Mean ± SD	Min	Max	CVb (%)
Muscle size				
Q_VOL_(cm^3^)	1833 ± 263	1254	2573	14.4
QACSA_MAX_ (cm^2^)	90.0 ± 12.5	67.6	125.0	13.9
Q_EFF_PCSA (cm^2^)	167.7 ± 18.8	125.9	205.1	11.2
Architecture				
QF_L_ (mm)	106.0 ± 10.2	74.5	126.5	9.6
QΘ_P_ (^o^)	15.7 ± 1.7	12.0	19.2	10.7
Joint mechanics				
PT_MA_ (cm)	4.2 ± 0.3	3.5	4.9	6.7

*indicates corrected *p* < 0.05, **indicates corrected *p* < 0.01

*Q*_*VOL*_ quadriceps muscle volume, *QACSAmax* quadriceps maximum anatomical cross-sectional area, *Q*_*EFF*_*PCSA* quadriceps effective physiological cross-sectional area, *QF*_*L*_ quadriceps weighted fascicle length (mm) and *QΘ*_*P*_ Pennation Angle (^°^), *PTMA* patella tendon moment arm

**Table 2:** Descriptive statistics for the size and architecture of individual constituent quadriceps muscles (*n* = 53)

Muscle	Volume (cm^3^)	ACSA_MAX_ (cm^2^)	_EFF_PCSA (cm^2^)	FL (mm)	Θp (°)
VM	441.4 ± 67.9^b,c,d^	24.6 ± 4.3^b,c^	40.5 ± 7.6	106.1 ± 15.3	19.2 ± 3.8^c,d^
VI	546.9 ± 104.0^a,c,d^	25.2 ± 4.3^a,c,d^	52.7 ± 9.1^c^	101.2 ± 8.2^c^	12.9 ± 2.6^c^
VL	609.8 ± 98.3^a,b,d^	27.6 ± 4.9^a,b,d^	53.0 ± 8.2^b^	111.3 ± 11.5^b^	15.9 ± 2.6^a,b,d^
RF	240.1 ± 46.7^a,b,c^	12.7 ± 2.4^b,c^	21.5 ± 3.4	108.8 ± 14.6	13.6 ± 2.5^a,c^

Significant differences (*p* < 0.05) are denoted by ^a^different from VM, ^b^different from VI, ^c^different from VL and ^d^different from RF. Data are mean ± std

*ACSA*_*MAX*_ maximum anatomical cross-sectional area, _*EFF*_*PCSA* effective physiological cross-sectional area, *F*_*L*_ fascicle length (mm) and *Θ*_*P*_ Pennation Angle (°)

### Determinants of voluntary explosive torque

Early phase voluntary explosive torque (i.e., T_50_) was unrelated to any measure of quadriceps muscle architecture, size or moment arm. For QF_L_ there were no relationships with any explosive strength measures expressed in either absolute or relative terms (*r* ≤ 0.282; *p* ≥ 0.056; Table 3).

**Table 3:** Pearson’s product moment correlation coefficient (*r*-values) between musculoskeletal variables (muscle size and architecture, and moment arm) and explosive strength measures, specifically absolute and relative (to MVT) torque and sequential rate of torque development (RTD, Nm.s^−1^ and %MVT.s^−1^) of specific time periods, during explosive voluntary isometric contractions of the knee extensors (*n* = 53)

	Voluntary explosive torque									
	Absolute (Nm)					Relative (%MVT)				
	T_50_	T_100_	T_150_	RTD_50–100_	RTD_100–150_	T_50_	T_100_	T_150_	RTD_50–100_	RTD_100–150_
Muscle size										
Q_VOL_ (cm^3^)	0.205	0.495**	0.653**	0.567**	0.596**	− 0.062	− 0.126	− 0.122	− 0.119	0.079
QACSA_MAX_ (cm^2^)	0.208	0.544**	0.690**	0.644**	0.580**	− 0.050	− 0.042	0.010	0.002	0.131
Q_EFF_PCSA (cm^2^)	0.126	0.432**	0.589**	0.539**	0.557**	− 0.217	− 0.166	− 0.163	− 0.090	− 0.099
Architecture										
QF_L_ (mm)	0.042	0.201	0.218	0.282	0.104	− 0.011	0.055	0.069	0.104	0.000
QΘ_P_ (^o^)	0.210	0.336*	0.356*	0.294*	0.185	0.074	0.105	0.155	0.068	0.053
Joint mechanics										
PT_MA_ (cm)	0.172	0.263	0.306*	0.272	0.207	0.068	0.047	0.052	− 0.021	− 0.014

*indicates corrected *p* < 0.05, **indicates corrected *p* < 0.01

*Q*_*VOL*_ quadriceps muscle volume, *QACSA*_*MAX*_ quadriceps maximum anatomical cross-sectional area, *Q*_*EFF*_*PCSA* quadriceps effective physiological cross-sectional area, *QF*_*L*_ quadriceps weighted fascicle length, *QΘ*_*P*_ quadriceps weighted pennation angle, *PTMA* patella tendon moment arm, *RTD* rate of torque development

**Fig. 4:**
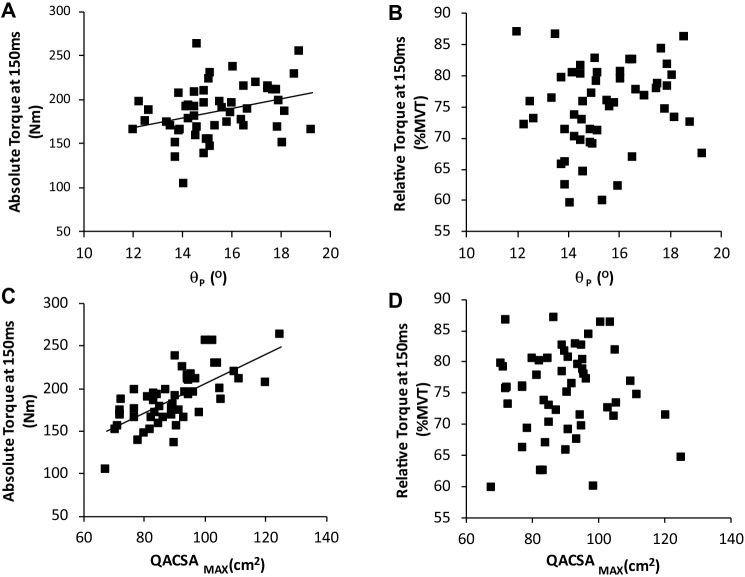
Relationship between QΘ_P_ (^o^) (**a** & **b**) and QACSA_MAX_ (**c **& **d**) and Absolute and Relative (%MVT) torque developed at 150 ms from torque onset during explosive voluntary contractions of the knee extensors (*n* = 53)

However, QΘ_P_ was weakly related to late phase explosive torque (T_100_ and T_150_; *r* = 0.336–0.356, *P* < 0.005; Fig. 4a) and RTD_50–100_ (*r* = 0.294, *p* = 0.04), but not RTD_100–150_ (*r *= 0.185, *p* = 0.208). When expressed relative to MVT, there were no relationships between any phase of explosive torque / sequential RTD and mean QΘ_P_ (*r* ≤ 0.155; *p* ≥ 0.39) (Fig. 4b).

There were moderate relationships between muscle size measures (Q_VOL_, QACSA_MAX_ and Q_EFF_PCSA) and absolute explosive torque, during the later phase of explosive contractions (*r* = 0.432–0.690, All *p* < 0.007; Fig. 4c), in all cases being stronger for T_150_ than T_100_. Middle and late phase sequential RTD (i.e., RTD_50–100_ and RTD_100–150_) expressed in absolute terms were also moderately related to muscle size variables (*r* = 0.539–0.644, All *p* < 0.001). However, when torque was calculated relative to MVT there were no relationships observed between muscle size and any measure of explosive torque/sequential RTD (*r* ≤ 0.136; *p* ≥ 0.30; Table 3*; *Fig. 4d).

PT_MA_ was weakly related to absolute T_150_ only (*r* = 0.31, *p* = 0.026), but once explosive strength was expressed relative to MVT no relationships were observed (*r* ≤ 0.104; *p* ≥ 0.402; Table 3).

### Determinants of evoked explosive (octet) torque

Evoked T_50_ was very weakly related to QΘ_P_ (*r* = 0.295, *p* = 0.04), but not QF_L._ All three indices of muscle size were moderate-strongly related to absolute evoked T_50_ (Q_VOL_, QACSA_MAX_ and Q_EFF_PCSA; *r* = 0.641–0.781, *P* < 0.001; Table 4) and these relationships were stronger than the relationships with any voluntary measures of explosive strength irrespective of time point/phase. Evoked explosive torque was also unrelated to moment arm. When expressed relative to MVT, there were no relationships between evoked explosive torque and any musculoskeletal variables.

**Table 4:** Pearson’s product moment correlation coefficient (*r*-values) between musculoskeletal variables (muscle size and architecture, and moment arm) and involuntary explosive strength measures, specifically absolute and relative (to MVT) torque at 50 ms (T_50_) during evoked octet isometric contractions of the knee extensors (*n* = 53)

	Absolute (Nm)	Relative to MVT (%)
Muscle size		
Q_VOL_ (cm^3^)	0.781**	− 0.211
QACSA_MAX_ (cm^2^)	0.696**	− 0.233
Q_EFF_PCSA (cm^2^)	0.641**	− 0.072
Architecture		
QF_L_ (mm)	0.179	− 0.056
QΘ_P_ (^°^)	0.295*	− 0.064
Joint mechanics		
PT_MA_ (cm)	0.260	− 0.130

*indicates corrected *p *< 0.05, **indicates corrected *p* < 0.01

*Q*_*VOL*_ quadriceps muscle volume, *QACSAmax* quadriceps maximum anatomical cross-sectional area, *Q*_*EFF*_*PCSA* quadriceps effective physiological cross-sectional area, *QF*_*L*_ quadriceps weighted fascicle length, *QΘ*_*P*_ quadriceps weighted pennation angle, *PTMA* patella tendon moment arm

### Musculoskeletal contribution to voluntary and evoked (octet) torque

Multiple regression analysis for the combined influence of musculoskeletal variables, revealed that there only one variable, Q_VOL_, contributed to the explained variance in voluntary T_100_ (34%) and T_150_ (54%), as well as evoked T_50_ (63%).

## Discussion

This study assessed the relationships between in vivo muscle and joint morphology and explosive knee extension strength (voluntary and evoked torque) in young men, observing positive relationships between θ_P_ (weak), muscle size (moderate to strong) and moment arm (weak) with absolute voluntary late phase explosive strength, but no associations of F_L_ and any measures of explosive strength. Furthermore, as there were no relationships between θ_P_, or any musculoskeletal factors, and relative expressions of explosive strength (i.e., once MVT was accounted for), these findings suggest that any influence of these musculoskeletal factors on explosive strength is via maximum strength rather than a direct and independent effect on explosive strength per se. As expected, muscle size measures had a more pronounced relationship with evoked than voluntary explosive strength, but surprisingly F_L_ and moment arm were unrelated to evoked explosive strength, and although θ_P_ was weakly associated with evoked explosive strength, this association was no greater than with voluntary explosive strength.

In a similar manner to our previous investigation (Folland et al. 2014), the current study found explosive strength measurements during the early phase of contraction were highly variable between individuals in this cohort of healthy young men (absolute T_50_, CVb 46%; relative T_50_, CVb 42%), but this variability progressively decreased as the contraction progressed. The important consequences of explosive strength for the performance of tasks where the time available to generate contractile torque is limited (e.g., sprinting (Tillin et al. 2013b), responding to perturbations of balance (Behan et al. 2018)) would appear to highlight the need to understand this variability in explosive strength. The current study involved comprehensive assessment of muscle architecture at ten sites throughout the quadriceps muscle, and deliberately used a long probe to minimize the amount of extrapolation required for F_L_ measurements. Our data for in vivo quadriceps muscle architecture are in general agreement with the range of values typically observed in similar cohorts (Blazevich et al. 2007; Ema et al. 2013; Strasser et al. 2013). However, our F_L_ measurements were longer than in some previous studies (Blazevich et al. 2006; Franchi et al. 2014), likely due to their muscle architecture measurements being done at a more extended knee joint angles and could also be due to the current study making more comprehensive measurements (ten sites) with minimal extrapolation. This comprehensive assessment in a large population of heterogeneous healthy young men revealed a surprisingly modest between individual variability in muscle architecture measurements (Θ_P_, CVb 11%; F_L_, CVb 10%), which may have limited the scope for identifying a relationship of these variables with explosive strength. For example, the standard deviation for Θ_P_ and F_L_ averaged throughout the quadriceps were < 2°and < 10 mm, respectively. Whilst other cohorts are known to have more distinct muscle architecture values (e.g., older adults or trained individuals) these cohorts exhibit multifactorial physiological differences (e.g., neural activation, muscle size, architecture and function) that preclude combining cohorts to isolate the relationship between muscle architecture and explosive strength.

In the current investigation there were no relationships between any of the musculoskeletal variables and early phase voluntary explosive strength measures (i.e., absolute or relative T_50_). This was contrary to our hypotheses that early phase explosive strength would be positively related to: (i) Θ_P_, due to the larger fascicle rotation of pennate fascicles, particularly at low forces (Brainerd and Azizi 2005); and (ii) F_L_, due to more sarcomeres in series and thus increased fascicle shortening velocity. These findings indicate that any effects of muscle architecture are insufficient to significantly effect in-vivo early phase explosive strength in a large cohort of young men. This appears a novel finding as to the authors’ knowledge no previous studies have examined the direct relationship of Θ_P_ and F_L_ with explosive strength. Contrary to our F_L_ findings, one study postulated a negative relationship between the changes in a surrogate measure of F_L_ (moment–angle relationship) and explosive strength after resistance training (Blazevich et al. 2009a), but as F_L_ was not directly measured in that study the value of this finding is unclear. Previous research has highlighted the importance of neural drive for early phase (i.e., 0–50 ms after contraction onset) isometric explosive strength expression (Folland et al. 2014; DelVecchio et al. 2019) and the predominant influence of neural drive may dwarf the role of any musculoskeletal variables. Early phase explosive strength has also been associated with the contractile response to an evoked twitch (Folland et al. 2014), but this may reflect calcium release from the sarcoplasmic reticulum in response to a single action potential rather than muscle morphology per se.

In the later phases of explosive torque production (i.e., absolute T_100_ and T_150_), the present study observed relationships with Θ_P_ (*r* = 0.336–0.356), all indices of muscle size (*r* = 0.432–0.690) and joint moment arm (*r* = 0.306), however no relationships were observed with F_L_. The correlations between muscle size indices and mid/late phase explosive strength was similar to our previous findings in other muscle groups (elbow flexor volume & force at 150 ms *r* = 0.69, (Erskine et al. 2014); knee flexor volume and time to 90 Nm of torque *r* = − 0.53, (Evangelidis et al. 2017)). Furthermore, the relationship of muscle size indices with mid/late phase explosive strength is consistent with the known increasing influence of MVT on explosive torque production as the contraction progresses (Andersen and Aagaard 2006; Folland et al. 2014), that was corroborated by the current experiment (T_50_, *r* = 0.327; T_100_, *r* = 0.502; T_150_ *r* = 0.86).

Multiple regression analysis of the combined musculoskeletal variables revealed that Q_VOL_ was the only independent determinant of late phase explosive strength. Furthermore, when voluntary explosive torque was expressed relative to MVT, it was unrelated to any of the musculoskeletal variables (*r* ≤ < 0.155). Taken together the regression analysis and relative explosive strength data indicate that Θ_P_ and moment arm may simply co-vary with Q_VOL_, and thus also MVT, rather than being independent predictors of explosive strength.

Evoked octet contractions bypass the voluntary nervous system to reveal the muscle–tendon unit’s capacity for explosive torque production and highlight the influence of musculoskeletal characteristics (de Ruiter et al. 2004; Folland et al. 2014). Muscle size indices (*r* = 0.641–0.781) and Θ_P_ (*r* = 0.295) were found to be related to evoked T_50_. However, when evoked torque was expressed relative to MVT there were no longer any relationships. Therefore, these musculoskeletal variables did not have an independent influence on evoked explosive strength when MVT was accounted for. We have previously observed similar findings when examining the relationship of explosive strength and muscle tendon unit stiffness (Massey et al. 2017). It therefore appears that the muscle and tendon characteristics are unlikely to play an important role in explosive torque independent of maximal torque production.

There are a number of limitations to the present study, muscle architecture measures were conducted at rest, and therefore any changes in Θ_P_ or F_L_ with contraction were unaccounted for. However, our muscle architecture measures were taken at the same knee joint angle used for the functional (strength) measurements to remove this common discrepancy between resting and functional joint angles. It was thought that a greater resting fascicle length in a standardised joint position (the optimum knee joint angle for torque production) would reflect more sarcomere in series. However, this resting fascicle length may not precisely reflect a standardised optimum sarcomere length compared to contraction (due to elongation of the series elastic component and concomitant shortening of the muscle fascicles during contraction), as well as variability in optimum sarcomere lengths between quadriceps muscles and participants, making it difficult to infer the number of sarcomeres in series from measurements of resting length as done in the present study. The resting architecture measurements in the current study would seem to be most closely related to the early phase (0–50 ms) of contraction when torque production was relatively low. Despite this, we found Θ_P_ to be related to late phase explosive strength (100 and 150 ms), which might suggest the possibility of a stronger correlation if architectural measurements were made at moderate to high torques.

The long (92 mm) probe used in this study minimised the requirement for extrapolation, and thus errors, within the F_L_ measurements (Franchi et al. 2018). There are inherent assumptions when using 2D B-Mode ultrasonography that the 2D image represents the architecture of a complex 3D structure. Future use of more advanced imaging techniques such as diffusion tensor imaging (DTI) to quantify 3D architecture may facilitate a more comprehensive evaluation of the importance of muscle architecture for explosive strength and overall muscle function.

In conclusion, musculoskeletal variables showed no relationship with absolute early phase explosive voluntary torque (≤ 50 ms), but θ_P_ (weak), muscle size (moderate to strong) and moment arm (weak) were related to late phase explosive voluntary torque (≥ 100 ms). The surprisingly consistent muscle architecture values in this cohort, whilst reflective of a heterogenous population of healthy young men, may have limited the scope for architecture to be related to explosive strength. Explosive strength expressed relative to MVT (i.e., once MVT was accounted for) was unrelated to any musculoskeletal variables. Therefore, it seems likely that these morphological factors are related to late phase absolute explosive voluntary torque via MVT, rather than as independent determinants.

## Abbreviations

CVb: Coefficient of Variation between subjects
F_L_: Mean Quadriceps Fascicle Length
MVT: Maximal Voluntary Torque
PT_MA_: Patella Tendon Moment Arm
QACSA_max_: Sum of maximal anatomical cross-sectional areas from quadriceps muscles
Q_EFF_PCSA: Effective Physiological cross-sectional area
Q_VOL_: Quadriceps muscle volume
Θ_P_: Mean Quadriceps Pennation Angle
RTD: Rate of Torque Development
T_50_: Torque at 50 ms
T_100_: Torque at 100 ms
T_150_: Torque at 150 ms
